# Comparing Classic and Interval Analytical Hierarchy Process Methodologies for Measuring Area-Level Deprivation to Analyze Health Inequalities

**DOI:** 10.3390/ijerph15010140

**Published:** 2018-01-16

**Authors:** Pablo Cabrera-Barona, Omid Ghorbanzadeh

**Affiliations:** 1 Department of Geoinformatics—Z_GIS, University of Salzburg, Schillerstraße 30, 5020 Salzburg, Austria; omid.ghorbanzadeh@stud.sbg.ac.at; 2 Instituto de Altos Estudios Nacionales, Av. Amazonas N37-271 y Villalengua, Quito 170507, Ecuador

**Keywords:** deprivation, analytical hierarchy process, self-reported health, self-reported quality of life, inequality

## Abstract

Deprivation indices are useful measures to study health inequalities. Different techniques are commonly applied to construct deprivation indices, including multi-criteria decision methods such as the analytical hierarchy process (AHP). The multi-criteria deprivation index for the city of Quito is an index in which indicators are weighted by applying the AHP. In this research, a variation of this index is introduced that is calculated using interval AHP methodology. Both indices are compared by applying logistic generalized linear models and multilevel models, considering self-reported health as the dependent variable and deprivation and self-reported quality of life as the independent variables. The obtained results show that the multi-criteria deprivation index for the city of Quito is a meaningful measure to assess neighborhood effects on self-reported health and that the alternative deprivation index using the interval AHP methodology more thoroughly represents the local knowledge of experts and stakeholders. These differences could support decision makers in improving health planning and in tackling health inequalities in more deprived areas.

## 1. Introduction

Area-level measures of deprivation are important tools for quantifying multidimensional social and material disadvantages and for studying health inequalities [1,2,3,4]. The motivations for developing these kinds of indices are grounded on the need of tackling inequality. For this reason, these indices are also tools that support decision-making processes aimed at the improvement of people’s health and quality of life. 

There are several kinds of deprivation indices that use different indicators and are constructed in different ways. For instance, the Townsend score [5,6] is a material deprivation index composed of just four variables. The Townsend score is obtained by the summation of the standardized scores for each variable. The Carstairs deprivation index [7] is very similar to the Townsend index and is also constructed as the combination of unweighted standardized variables. The Index of Multiple Deprivation 2000 encompasses several kinds of indicators, including indicators of mortality, disability, and healthcare accessibility [8,9]. There are other indices that have been constructed by applying statistical methods such as the principal components analysis [3,10]. Furthermore, the principal components analysis has been shown to be a suitable technique to weight the indicators that compose a deprivation index [11]. Deprivation indices can use individual-level and census-level indicators, and these indicators can be weighted by using regression model coefficients as weights [12]. Expert-based weights can also be used to construct deprivation indices [13].

Thus, a key issue in the calculation of any deprivation index is to determine how to weight the different indicators that comprise the index. The weights determine the trade-offs between the different dimensions of a multidimensional index [14], and changes applied to these weights may influence the outcomes of deprivation representations in a study area and, consequently, influence decision-making processes. When using expert-based weights, multi-criteria decision analysis (MCDA) methods are effective techniques because they can diminish bias when incorporating subjective information such as experts’ judgments. Additionally, these methods are useful in decision making and planning and can be incorporated in geographic information system (GIS) environments to support spatial multi-criteria analyses of deprivation areas [1,13]. For instance, the order weighted average (OWA) is an MCDA method that has been used to validate deprivation indices that use qualitative information [13], as well as to create alternative scenarios of a deprivation index [1,15]. The OWA method uses a fuzzy logic that is used to rank the indicators based on their values. However, OWA works with ordered weights, weights that are based on indicators’ weights that have been previously calculated with other techniques. An MCDA method that is used to calculate indicators’ weights to construct deprivation indices is the analytical hierarchy process (AHP) [1,16]. 

The AHP developed by Saaty [17] is one of the most popular methods of MCDA. What is more, the AHP has been applied to weight indicators of deprivation indices in GIS environments [1]. Integration of AHP and GIS offers a useful and practical approach to decision-making processes [18] within a collaborative and spatial framework [19,20]. The AHP supports the creation of weights based on experts’ judgments structured in a pairwise comparison matrix [21,22] by applying Saaty’s scale of importance intensities (scale of 1 to 9). In the case of a deprivation index, these intensities represent the experts’ judgments regarding which indicators of the index contribute more to deprivation in comparison to the rest of the indicators. Despite the advantages of the AHP, this method is subject to some criticism. Since the comparison of indicators is based on subjective judgments, some degree of uncertainty and inconsistency may occur [23]. Additionally, some experts may not be totally aware of the nature and importance of some of the indicators considered [24]. The uncertainty and inconsistency may be exacerbated when experts and stakeholders analyze social variables beyond variables related to physical geography and environment.

Several methods have been applied to diminish uncertainty and inconsistency associated with the AHP approach, such as the application of fuzzy logic, the use of OWA, and the proposal of spatial sensitivity analysis [18,21,25]. With these kinds of methods, the different indicators can be aggregated considering their different values in the different zones of the study area. They also can capture the most relevant qualitative and quantitative information between the different indicators. However, experiences in applying MCDA methods, such as AHP and OWA, to construct deprivation indices are not common in scientific literature. 

This outlined background situation highlights two key issues, namely, (1) the development of deprivation indices applying AHP has not been widely explored by the scientific community, and (2) the AHP is subject to the uncertainty associated with the experts’ judgments and to the limitations of the Saaty scale: diverse experts’ judgments are combined to only one value of the scale, which may not totally represent the experts’ disagreements with respect to the pairwise comparison of indicators. 

The purpose of this paper is to apply an interval AHP to the calculation of a deprivation index and to compare the result obtained to an index created by applying the traditional AHP method. To compare both indices, we compare their indicators’ weights, the spatial representations of the indices, and the influence of the indices on a health outcome (self-reported health). 

The interval AHP is used to improve the accuracy of the indicators’ weights that compose the deprivation index. The interval AHP calculates the weightings through interval pairwise comparison matrices, which means applying a more flexible and accurate AHP. The deprivation index chosen for the study is the multi-criteria deprivation index for the city of Quito [15,16]. This index was constructed using AHP to weight its indicators and has been proven to explain the individual-level variations in health outcomes, to some degree. Further, a second interval AHP method that uses an interval pairwise comparison matrix [24] was also applied in this research. 

## 2. Methods 

### 2.1. The Multi-Criteria Deprivation Index for the City of Quito Using Traditional AHP 

**Figure 1:**
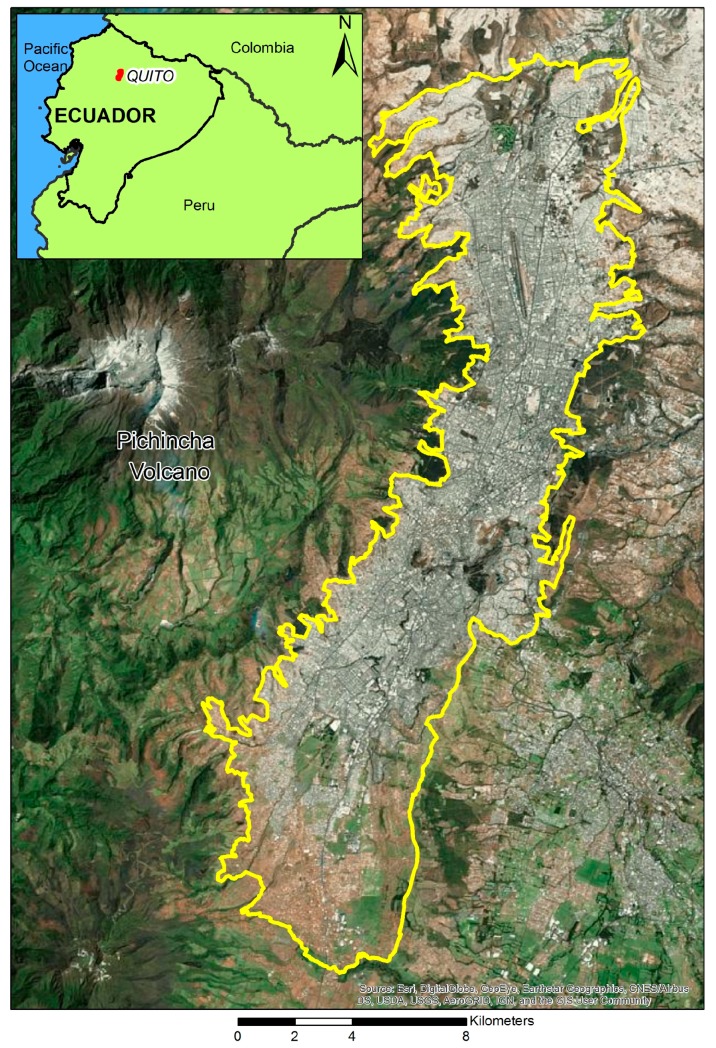
The city of Quito, Ecuador.

Quito is the capital of Ecuador, located in the northern Andes region of the country (Figure 1). The city is part of the Metropolitan District of Quito, an administrative area where more than 2 million people live. Most of the population of the Metropolitan District of Quito live in the city of Quito, which is home to more than 1.6 million inhabitants, over 80% of whom are *mestizo* (mixed-ethnicity, generally indigenous-white). The city consists of 34 urban parishes. However, the smallest areal unit that the city can be divided into is the census block. The city of Quito comprises 4036 census blocks. The census blocks’ population average is 398.25, standard deviation 156.20. The multi-criteria deprivation index for the city of Quito (MDIQ) is calculated at the census block level because the indicators used for this index are extracted from the Ecuadorean Population and Housing Census.

The MDIQ is an index proposed by [16] with two main characteristics: (i) its indicators are based on a human rights approach that considers the satisfaction of basic needs as a fundamental prerequisite for achieving a better quality of life [1,26,27] and, thus, deprivation can be expressed as the restriction of these human rights-based conditions; and (ii) its indicators are weighted by applying the AHP based on the experts’ criteria. The indicators to construct the MDIQ were chosen considering the Good Living conceptual framework, which states that having access to services that satisfy basic human needs such as education, health, and proper living conditions is the foundation to construct cohesive societies of Good Living [1]. The chosen indicators also have an affinity to material and social deprivation, as documented in previous studies [1,3,10,28]. All in all, we define deprivation as the limited access to services that satisfy basic human needs and rights. The indicators of the MDIQ are shown in Table 1.

The AHP [17] is a method that structures complex decision problems into a hierarchical system by applying a pairwise comparison matrix for the considered indicators using experts’ judgments to assign weights to these indicators. The pairwise comparison matrix is constructed by using scores that represent experts’ judgments when comparing the importance of each indicator in relation to all the other indicators. In the case of the indicators that make up the MDIQ, 13 experts were consulted. The experts were professionals and decision-makers from the fields of geography, health, and urban studies. The scores for the indicators’ pairwise comparisons range from 1 to 9, where 1 means ‘equal importance to’ and 9 means ‘enormously more important than’. Intermediate values (2, 4, 6, 8) and reciprocals (inverse values) can also be used in the pairwise comparison matrix. For instance, the value of 3 located in row B (Table 2) means that indicator B is moderately more important than indicator A. An advantage of the AHP is that it is possible to test the consistency of the pairwise comparison matrix. This consistency is calculated by means of the consistency ratio (CR). The CR is the ratio between the consistency index (CI) and the random index (RI). The CI is a function of the weights’ vector, the pairwise comparison matrix, and the number of indicators used. The RI represents the consistency index of a random pairwise comparison matrix. A CR value lower than 0.10 indicates that there is consistency in the pairwise comparison and that the obtained weights can be considered as reliable [21]. For a full description of how the AHP works, please see [21,29]. For a full description of how the AHP method is applied to construct the MDIQ, please see [16]. The AHP pairwise comparison matrix that was used to construct the MDIQ and the AHP-based weights of the indicators of the MDIQ are shown in Table 2. 

Table 2 shows that the indicators with the highest weights are the percent of the population that works in unpaid jobs, percent of households without access to drinking water from the public system, and the distance to the nearest primary healthcare service. The CR for the MDIQ weighting was 0.038, a value lower than 0.10, which means that the weights calculated are consistent enough to be used to construct an index. The MDIQ is calculated by adding the weighted normalized indicators. The indicators are normalized by applying the min-max normalization [16]. 

To overcome disagreements between experts, the arithmetic mean method can be applied [29], as was the case in the MDIQ. However, experts’ pairwise comparisons of the applied indicators can vary. Thus, variations in judgments are not fully represented in the final weights of the traditional AHP. In other words, the pairwise comparison matrix of the traditional AHP only shows crisp numbers to represent the experts’ judgments. For this reason, the interval pairwise comparison matrix (IPCM) method was applied to create alternative indicators’ weights than the weights calculated with the traditional AHP method. 

### 2.2. The Interval AHP: Applying the Interval Pairwise Comparison Matrix 

The interval pairwise comparison matrix (IPCM) method [24] was applied to create alternative weights for the MDIQ. Although the traditional AHP is a common method for decision-making purposes, nearly all researchers think that outputs of the method are significantly influenced by the experts’ judgments [24]. Using crisp numbers that correspond to the experts’ judgments may result in inaccurate weightings. In the traditional AHP, several experts are asked to compare indicators. Thus, it is very likely to get a diversity of judgments. In addition, sometimes experts do not agree with each other or they are not even sure about their own judgment within a number. To overcome these kinds of problems, intervals and not crisp numbers can be used to represent the experts’ judgments. The IPCM method was used in this study to extract new weights from the experts’ judgments. The IPCM method is based on the precedent that the Saaty scale-based values used in the AHP can be expressed in intervals rather than by a specific crisp value. Hence, the pairwise comparison X can be expressed as:

(1) $$X=\;\left[l_{ij},u_{ij}\right]$$

where $l_{ij}$ and $u_{ij}$ denote the lower and upper limits of interval X [30], which shows that the indicator $x_{i}$ is between $l_{ij}$ and $u_{ij}$ times as preferred as the indicator $x_{j}$, and all of the interval elements are organized in the matrix A [24,31]:

(2) $$A=\left[\begin{array}{cc} \begin{array}{c} 1\\ \left[l_{21},u_{21}\right]\\ \begin{array}{c} \vdots \\ \left[l_{n1},u_{n1}\right] \end{array} \end{array} & \begin{array}{c} \left[l_{12},u_{12}\right]\\ 1\\ \begin{array}{c} \vdots \\ \left[l_{n2},u_{n2}\right] \end{array} \end{array} \end{array}\begin{array}{cc} \begin{array}{c} \ldots \\ \ldots \\ \begin{array}{c} \vdots \\ \ldots \end{array} \end{array} & \begin{array}{c} \left[l_{1n},u_{1n}\right]\\ \left[l_{2n},u_{2n}\right]\\ \begin{array}{c} \vdots \\ 1 \end{array} \end{array} \end{array}\right]$$

where, $\;l_{ij}\geq 0,\;u_{ij}\geq 0$, ∀ *i,j* = 1, 2, …, *n*. Notice that if $l_{ij}=u_{ij}$, ∀ *i,j* = 1, 2, …, *n*, matrix A turns into the pairwise comparison matrix of the traditional AHP. In matrix A, every element has two conditions: $l_{ij}=u_{ij}\;$or $l_{ij}\leq u_{ij}$, ∀ *i,j* = 1, 2, …, *n*. This means that experts are allowed to compare any pair within the interval related to a crisp value. 

The matrix A can be expressed in two different matrices, B and C, and we can say that matrix A has a reasonable CR when both matrices B and C have reasonable CRs [24,31]. This means that A is a consistent pairwise comparison matrix when $0<{\mathrm{CR}}_{\mathrm{matrix}\;B}<0.1\;$and $0<{\mathrm{CR}}_{\mathrm{matrix}\;C}<0.1$.

A statistical model of the Monte Carlo simulation (MCS) was applied for calculating the crisp weightings based on the IPCM outcomes. MCS is a common approach for statistical sampling, especially in complex systems [32]. Using MCS, an average value is calculated as a definite number through a repeated number of random statistical samples between lower and upper limits of an interval [33]. The pairwise comparison matrices produced had a reasonable CR, and the sum of all the final weights obtained was equal to one. These new weights were applied to calculate a new deprivation index that we call ‘interval MDIQ’ or I-MDIQ.

### 2.3. Comparison and Validation of the MDIQ and I-MDIQ 

To compare and validate both the MDIQ and the I-MDIQ, the influence of these indices on self-reported health was evaluated. Deprivation influences health outcomes [3,4,34] including self-reported health [15,35,36]. Thus, regression models can be used to model the relationship between deprivation and self-reported health. For this study, two types of models are suitable for use: logistic generalized linear models (dependent categorical variable of self-reported health) and multilevel models (individual-level dependent variable of self-reported health spatially nested in an area-level variable of deprivation). In the models, two independent variables are considered: deprivation (either MDIQ or I-MDIQ) and self-reported quality of life. The latter variable was used as a controlling individual-level independent variable for the models, considering that individual quality of life is an important variable that influences individual health [37]. The measures of self-reported health and self-reported quality of life were extracted from 489 responses obtained from a survey carried out in Quito in 2014. For details of the survey design, see [38]. The margin of this survey sampling error was ±4 with a level of confidence of 95%. Self-reported health and self-reported quality of life were measured using a 1–5 Likert scale whereby, in the case of the self-reported health, a value of 5 indicates having excellent health and a value of 1 indicates having very bad health; and, in the case of self-reported quality of life, a value of 5 indicates being very satisfied with one’s quality of life and a value of 1 means being very unsatisfied with one’s quality of life. 

The logistic generalized linear model can be expressed as:

(3) $$\mathrm{SRH}=\mathrm{logit}(\beta_{1}+\beta_{2}\mathrm{SRQoL}+{\;\beta }_{3}D)$$

where SRH is the self-reported health, SRQoL is the self-reported quality of life, and D represents either MDIQ or I-MDIQ. 

The multilevel model can be expressed as a conditional growth equation:

(4) $${\mathrm{SRH}}_{ij}=\beta_{0j}+\beta_{1j}{\mathrm{SRQoL}}_{ij}+r_{ij}$$

where ${\mathrm{SRH}}_{ij}$ is the self-reported health, ${\mathrm{SRQoL}}_{ij}$ is the self-reported quality of life, and $r_{ij}$ represents the error at the individual level. The individual-level intercept and slope can be expressed as:

(5) $$\beta_{0j}={\;\gamma }_{00}+\gamma_{01}D_{j}+\mu_{0j}$$

(6) $$\beta_{1j}={\;\gamma }_{10}+\gamma_{11}D_{j}+\mu_{1j}$$

where $D_{j}$ represents the area-level variable of the model (either MDIQ or I-MDIQ) and the random terms $\mu_{0j}$ and $\mu_{1j}$ are the errors at the area level. 

To compare and validate the MDIQ and the I-MDIQ based on the regression models used, two statistics were compared: the slope index of inequality obtained from the logistic generalized linear modeling, and the variation partition coefficient obtained from the multilevel modeling. The slope index of inequality is the slope coefficient of a regression where, in the case of deprivation in relation to health, the larger the slope coefficient, the greater the impact of deprivation on a health outcome [39] such as self-reported health. For this study, the coefficient considered corresponds to the MDIQ or I-MDIQ. The variation partition coefficient is useful for evaluating the variances of an individual-level measure, such as self-reported health, in relation to an area-level measure such as a deprivation index [16].

## 3. Results

Table 3 shows the interval comparison matrix obtained. The indicators with the highest weights are the percent of the population that has no public social/health insurance, percent of the population that works in unpaid jobs, and the percent of households without access to drinking water from the public system. Thus, these are the three most important indicators of the I-MDIQ. The obtained CRs for the I-MDIQ weighting were 0.047 and 0.048, both of them lower than 0.10. This means that the whole process of the I-MDIQ is consistent and the results are acceptable.

**Figure 2:**
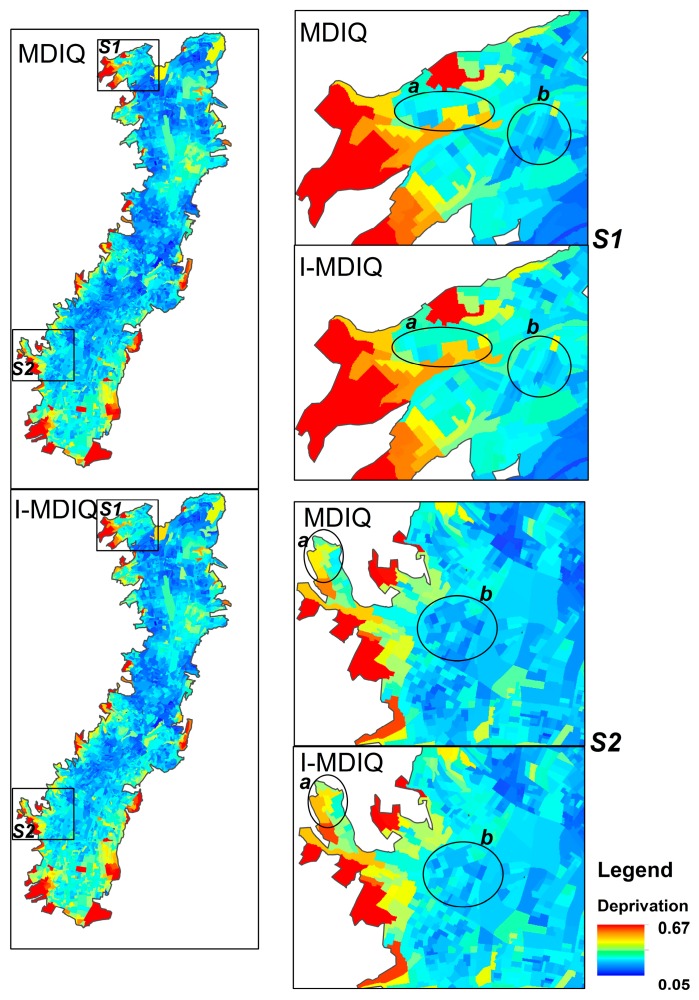
The MDIQ and the I-MDIQ at the census block scale. In general, both indices are virtually equivalent. However, differences can be identified in several census blocks: S1 and S2 are example areas where zones a and b depict differences between the indices. I-MDIQ presents some census blocks with higher deprivation levels than MDIQ.

Figure 2 depicts the MDIQ and I-MDIQ expressed at the finest scale available for the study area, the census block scale. Both indices—MDIQ and I-MDIQ—represent similar deprivation patterns in the city of Quito: the more deprived areas (red color range) are located in the peripheries of the city. However, there are small differences when it comes to the individual analysis of some of the census blocks. For example, in Figure 2, two examples are shown: S1 and S2, each one with two sample zones, a and b. In the case of S1 sample a, a yellow-colored census block in the MDIQ turns orange in the case of the I-MDIQ. In the case of S1 sample b, the blue colors in the MDIQ are darker than the blue colors corresponding to the I-MDIQ. In the case of S2 sample a, a yellow-colored census block in the MDIQ turns orange in the case of the I-MDIQ. In the case of S2 sample b, the blue colors in the MDIQ are darker than the blue colors corresponding to the I-MDIQ. 

This means that in some census blocks, the I-MDIQ shows higher deprivation values than the deprivation values of the MDIQ. 

Table 4 shows the results of the applied logistic generalized linear and multilevel models. The slope indices of inequality and the *t*-values are the results obtained from the logistic generalized linear models. The variation partition coefficients and the likelihood ratio tests are the results obtained from the multilevel models. The slope index of inequality is slightly higher in the case of the MDIQ. However, this difference is not marked, and it is also important to mention that both slopes were not found to be significant because their *t*-values were below 1.96, which is the critical threshold value at the 5% level of significance. Additionally, in the logistic model considering the MDIQ, the odds ratios’ confidence intervals (95% of confidence) were 0.02 and 4475.65 for the slope index of inequality, while in the logistic model considering the I-MDIQ, the odds ratios’ confidence intervals were 0.02 and 2425.43 for the slope index of inequality. These values corroborate the non-significance of both slope indices of inequality. However, the self-reported quality of life was found to be significant in the logistic generalized linear models performed (*t*-value of 2.04 and odds ratios’ confidence intervals between 1.01 and 1.69 for both models).

The variation partition coefficients of both indices are also equivalent. The likelihood ratio tests showed that the variation partition coefficients were significant at the 5% level of significance because both tests’ values were larger than 3.84 (the 5% point of a chi-squared distribution on 1 degree of freedom is 3.84). The variation partition coefficient’s value obtained for the case of the MDIQ was higher than the value obtained for the case of the I-MDIQ. This means that the original MDIQ is more significant when variances of self-reported health are attributed to differences between deprivation areas. In other words, the neighborhood effects of the MDIQ were found to be more significant than the neighborhood effects of the I-MDIQ.

## 4. Discussion

This research aimed to apply an interval AHP methodology to the calculation of a deprivation index and to compare the result obtained to an index created by applying the classic AHP method. The interval AHP is a novel MCDA approach, which, as well as the traditional AHP method, supports a spatially explicit analysis of deprivation. The MCDA techniques have been broadly and successfully used for decision-making processes by allocating the elements of a problem to several hierarchical levels [33,40]. Even though the AHP is an approach that has been used for weighting indicators of deprivation indices [1,16], this approach is vulnerable to experts’ misjudgments. When comparing social indicators in a pairwise comparison matrix, disagreements between experts’ judgments may occur, mainly because these kinds of indicators are related to the very complex and multidimensional concept of quality of life, which most researchers accept is not easily defined [37]. We minimized the problem of experts’ disagreement by optimizing the AHP with the interval comparison pairwise matrix. The flexibility of the novel approach expedites the construction of a pairwise comparison matrix that better represents the diversity of experts’ judgments. The intervals expressed in the interval pairwise comparison matrix represents this diversity in judgment: differences of criteria between experts and variations of the own criteria of each expert. 

The MDIQ is a useful measure of deprivation associated with health inequalities [16]. Compared to the MDIQ, the new deprivation measure I-MDIQ is just as effective in capturing deprivation and health inequalities. The advantage of I-MDIQ is that the AHP-based weighting considers intervals that better represent the insights of local experts and stakeholders. Moreover, one of the indicators that received the highest weight is the ‘percent of the population that has no public social/health insurance’. This means that local experts considered this indicator as one of the most influential in terms of causing deprivation. The census of the city of Quito shows that a large proportion of the population has no public social/health insurance. This finding means that local experts’ judgments do reflect the reality of the study area and that the I-MDIQ could better represent the experts’ views. Additionally, experts’ judgments reflect the understanding that having health insurance is a critical issue that influences health and the quality of life. Indeed, it has been identified that having health insurance significantly impacts health, wellbeing, satisfaction with healthcare, and the accessibility to healthcare in terms of people’s perceptions [37,38]. Thus, assigning a higher weight to the indicator related to health insurance, as is the case in I-MDIQ, considers the importance of this social indicator when linking deprivation, health, and quality of life. In conclusion, it can be said that the interval pairwise comparison matrix used for the I-MDIQ better captures the experts’ judgments, and this information is useful in obtaining weights that are more fitted to reality. The I-MDIQ also shows higher deprivation in some census blocks. However, in general, both indices can also be considered equivalent. First, their values range from 0.05 to 0.67. Second, the results of the performed models are also similar for both indices. 

When it comes to analyzing the logistic generalized linear models and multilevel models that were applied, results show only minor differences. The slope index of inequality is similar for both indices MDIQ and I-MDIQ, although it is slightly greater for the MDIQ. Nevertheless, the slope indices of inequality were not found to be significant. It is important to mention that in the logistic generalized linear models the self-reported quality of life was found significant. This result suggests that the logistic generalized linear model is useful for associating two individual-level variables but this model may not describe the multilevel (multiscale) associations between the area-level measures of deprivation and the individual-level measures of self-reported health and self-reported quality of life. The multilevel models are useful tools to describe these associations. 

In the multilevel models, significant variances of self-reported health were found. In other words, the MDIQ and the I-MDIQ represent deprivation that influences self-reported health. Specifically, the obtained results suggest that 41% of the self-reported health variance is attributed to differences of zones expressing the MDIQ, and the 44% of the self-reported health variance is attributed to differences of zones expressing the I-MDIQ. However, when comparing both indices, the MDIQ showed more significant effects on self-reported health. 

The lack of significance of the slope index of inequality and the important significance of the variation partition coefficient suggest that the relationships between deprivation, self-reported health, and self-reported quality of life vary between places and that fitting a multilevel model that allows the relationships between variables to vary from place to place is a better option for analyzing the spatially hierarchical structure of individuals reporting their health and living in a specific context (census blocks expressing deprivation). 

The self-reported health reflects an individual’s integrated perception of health [41] that is not necessarily associated with objective measures of individual health but nevertheless is associated with more detailed measures of health status such as reported physical and mental health [42], and also to contextual objective measures of deprivation [15,39]. This means that self-reported health is an enduring concept that remains stable over time at the individual level but changes between groups of individuals living in different neighborhoods and that this change can respond to neighborhood-level characteristics such as deprivation. Indeed, it can be said that in the case of this study, variations in self-reported health can be attributed to differences between deprivation areas, and also attributed to differences in self-reported quality of life within these areas. In other words, how a person rates their health is associated with how this person rates their quality of life and to the multidimensional deprivation of their neighborhood. 

All in all, the MDIQ and the I-MDIQ had similar neighborhood effects on self-reported health. The advantage of using the I-MDIQ is that their weights better reflect experts’ judgments and local knowledge of the reality in the study area. However, in general terms, our findings suggest that both indices can be considered as equivalents. Decision-makers and planners could interchangeably use the MDIQ and the I-MDIQ if they aim to operate in a small scale (larger areas). In the case of smaller area interventions (such as interventions on a specific census block), decision-makers and planners need to take into consideration possible variations between the MDIQ and the I-MDIQ. 

Future research related to this work needs to extend the application of the classic AHP and the interval AHP to different indices of deprivation. This research gap is of great interest because the application of MCDA techniques to construct deprivation indices is not common, despite the importance of incorporating experts’ criteria and local knowledge in the development of more holistic and pluralistic measures of deprivation. For the public health domain, it may be interesting to relate the results of this study to additional individual-level subjective and objective variables, such as perceptions of social cohesion or income. Future studies may also use additional control variables (such as gender and age) in the regression models applied in this study. The AHP considers a top-down hierarchical structure assuming criteria as independent of each other. We propose that future research can apply the analytical network process (ANP) method in combination with the IPCM to construct deprivation indices. This method is based on the AHP but considers inner and outer influences and dependences of criteria in decision-making problems. An integrated approach of ANP and IPCM could extend the capability of analyzing multi-criteria and multidimensional deprivation and other socio-economic indices, with potentially important implications for decision making. 

## 5. Conclusions

The significance of this study lies in the introduction of an interval AHP method to weight indicators to construct social indices. We believe that traditional techniques can be improved by incorporating the interval pairwise comparison matrix to multi-criteria analyses of deprivation indices. Another contribution of this research is that we showed how the different applied AHP methods impact deprivation indices, which represent equivalent patterns of deprivation at the city level but different values of deprivation in several census blocks. This means that the I-MDIQ could be useful for analyzing deprivation in specific census blocks by considering more ‘realistic’ weights in the sense of more thoroughly representing the local knowledge of experts and stakeholders. On the other hand, we also found that variances of self-reported health, attributed to differences between deprivation areas, are similar in both indices: MDIQ and I-MDIQ. However, the neighborhood effects of the former were found to be more significant, which suggests that MDIQ is a consistent and robust measure of deprivation when studying health inequalities. 

## Figures and Tables

**Table 1:** Indicators used to construct the deprivation index.

Indicators
A: % of the population that have a long-term disability
B: % of the population that does not have any level of formal education or instruction
C: % of the population that has no public social/health insurance
D: % of the population that work in unpaid jobs
E: % of households with four or more persons per dormitory
F: % of households without access to drinking water from the public system
G: % of households without access to a sewerage system
H: % of households without access to the public electricity grid
I: % of households without garbage collection service
J: distance (meters) to the nearest primary healthcare service

**Table 2:** Pairwise comparison matrix, indicators’ weights, and the consistency ratio (CR) of the weights.

Indicator	A	B	C	D	E	F	G	H	I	J	Weights
**A**	1										0.048
**B**	3	1									0.067
**C**	3	2	1								0.090
**D**	2	2	2	1							0.111
**E**	1	1	1/2	1/2	1						0.039
**F**	4	4	3	3	6	1					0.228
**G**	2	2	1	1	4	1/2	1				0.102
**H**	2	1	2	1	4	1/3	2	1			0.108
**I**	1	1	1	1/2	3	1/3	1	1	1		0.076
**J**	2	2	1	1	3	1	1	2	2	1	0.131
CR = 0.038											

Note: The indicators’ codes correspond to the codes of Table 1.

**Table 3:** Pairwise comparison matrix, indicators’ weights, and the CR of the weights.

Indicator	A	B	C	D	E	F	G	H	I	J	Weights
**A**	1										0.0510
**B**	[3,5]	1									0.0881
**C**	[3,5]	[2,3]	1								0.1157
**D**	[2,3]	[1,2]	[1,2]	1							0.1080
**E**	1	1	[1/2,1]	1/2	1						0.0437
**F**	4	[3,4]	[2,3]	3	[5,6]	1					0.2175
**G**	[1,2]	[1,2]	[1/2,1]	[1/2,1]	[3,4]	1/2	1				0.0966
**H**	2	[1/2,1]	[1,2]	1	4	1/3	2	1			0.1052
**I**	[1/2,1]	[1/3,1]	[1/2,1]	1/2	[2,3]	1/3	[1/2,1]	1	1		0.0678
**J**	[1,2]	[1,2]	[1/2,1]	1	[2,3]	1	[1/2,1]	[1,2]	[1,2]	1	0.1073
CR_B_ = 0.0482; CR_C_ = 0.0474											

Note: The indicators’ codes correspond to the codes of Table 1.

**Table 4:** Statistical results of the applied models.

	Slope Index of Inequality	*t*-Value	Variation Partition Coefficient	Likelihood Ratio Test
MDIQ	2.14	0.67	0.41	15.03
I-MDIQ	1.85	0.61	0.44	7.61

Note: The slope index of inequality is the coefficient of the deprivation index in the logistic generalized linear model. The variation partition coefficient is obtained from the multilevel model dividing the variance of the intercept of the multilevel model by the total variance of this model. In the logistic generalized linear models, the self-reported quality of life was found to be significant.

## References

[1] Cabrera-Barona P., Murphy T., Kienberger S., Blaschke T. (2015). A multi-criteria spatial deprivation index to support health inequality analyses. Int. J. Health Geogr..

[2] Niggebrugge A., Haynes R., Jones A., Lovett A., Harvey I. (2005). The index of multiple deprivation 2000 access domain: A useful indicator for public health?. Soc. Sci. Med..

[3] Pampalon R., Hamel D., Gamache P., Raymond G. (2009). A deprivation index for health planning in Canada. Chronic Dis. Can..

[4] Havard S., Deguen S., Bodin J., Louis K., Laurent O., Bard D. (2008). A small-area index of socioeconomic deprivation to capture health inequalities in France. Soc. Sci. Med..

[5] Townsend P. (1987). Deprivation. J. Soc. Policy.

[6] Townsend P., Phillimore P., Beattie A. (1988). Health and Deprivation. Inequality and the North.

[7] Carstairs V., Morris R. (1989). Deprivation: Explaining differences in mortality between Scotland and England and Wales. Br. Med. J..

[8] Jordan H., Roderick P., Martin D. (2004). The Index of Multiple Deprivation 2000 and accessibility effects on health. J. Epidemiol. Community Health.

[9] Department of Environment, Transport and the Regions (DETR) (2000). Measuring Multiple Deprivation at the Small Area Level: The Indices of Deprivation 2000.

[10] Lalloué B., Monnez J.-M., Padilla C., Kihal W., Le Meur N., Zmirou-Navier D., Deguen S. (2013). A statistical procedure to create a neighborhood socioeconomic index for health inequalities analysis. Int. J. Equity Health.

[11] Cabrera-Barona P., Wei C., Hagenlocher M. (2016). Multiscale evaluation of an urban deprivation index: Implications for quality of life and healthcare accessibility planning. Appl. Geogr..

[12] Guillaume E., Pornet C., Dejardin O., Launay L., Lillini R., Vercelli M., Marí-Dell’Olmo M., Fernández Fontelo A., Borrell C., Ribeiro A.I. (2016). Development of a cross-cultural deprivation index in five European countries. J. Epidemiol. Community Health.

[13] Bell N., Schuurman N., Hayes M.V. (2007). Using GIS-based methods of multicriteria analysis to construct socio-economic deprivation indices. Int. J. Health Geogr..

[14] Belhadj B. (2012). New weighting scheme for the dimensions in multidimensional poverty indices. Econ. Lett..

[15] Cabrera-Barona P. (2017). Influence of Urban Multi-Criteria Deprivation and Spatial Accessibility to Healthcare on Self-Reported Health. Urban Sci..

[16] Cabrera-Barona P., Blaschke T., Gaona G. (2017). Deprivation, Healthcare Accessibility and Satisfaction: Geographical Context and Scale Implications. Appl. Spat. Anal. Policy.

[17] Saaty T.L. (1977). A Scaling Method for Priorities in Hierarchical Structures. J. Math. Psychol..

[18] Feizizadeh B., Shadman Roodposhti M., Jankowski P., Blaschke T. (2014). A GIS-based extended fuzzy multi-criteria evaluation for landslide susceptibility mapping. Comput. Geosci..

[19] Boroushaki S., Malczewski J. (2010). Measuring consensus for collaborative decision-making: A GIS-based approach. Comput. Environ. Urban Syst..

[20] Ghorbanzadeh O., Pourmoradian S., Blaschke T., Feizizadeh B. Nature Based Tourism Susceptibility Mapping by applying GIS-Decision Making Systems in East Azerbaijan Province, Iran. Proceedings of the 1st International Conference of SilkGIS.

[21] Boroushaki S., Malczewski J. (2008). Implementing an extension of the analytical hierarchy process using ordered weighted averaging operators with fuzzy quantifiers in ArcGIS. Comput. Geosci..

[22] Saaty R. (1987). The analytic hierarchy process—What it is and how it is used. Math. Model..

[23] Ho W. (2008). Integrated analytic hierarchy process and its applications—A literature review. Eur. J. Oper. Res..

[24] Feizizadeh B., Ghorbanzadeh O. GIS-based Interval Pairwise Comparison Matrices as a Novel Approach for Optimizing an Analytical Hierarchy Process and Multiple Criteria Weighting. Proceedings of the GI_Forum 2017.

[25] Chen Y., Yu J., Khan S. (2013). The spatial framework for weight sensitivity analysis in AHP-based multi-criteria decision making. Environ. Model. Softw..

[26] Mideros A. (2006). Ecuador: Defining and measuring multidimensional poverty. CEPAL Rev..

[27] Ramírez R. (2012). La vida buena como riqueza de las naciones. Rev. Ciencias Soc..

[28] Carstairs V. (1995). Deprivation indices: Their interpretation and use in relation to health. J. Epidemiol. Community Health.

[29] Bolloju N. (2001). Aggregation of analytic hierarchy process models based on similarities in decision makers’ preferences. Eur. J. Oper. Res..

[30] Sugihara K., Tanaka H. (2001). Interval Evaluations in the Analytic Hierarchy Process by Possibility Analysis. Comput. Intell..

[31] Liu F. (2009). Acceptable consistency analysis of interval reciprocal comparison matrices. Fuzzy Sets Syst..

[32] Jahanshahloo G., Hoseinzadeh F., Barzegarinegad A., Hatamaian H. (2012). Weight gain for interval pairwise matrices in the AHP with area of confidence by DEA method. Proceedings of the 4th National Conference of Data Envelopment Analysis.

[33] Feizizadeh B., Jankowski P., Blaschke T. (2014). A GIS based spatially-explicit sensitivity and uncertainty analysis approach for multi-criteria decision analysis. Comput. Geosci..

[34] Lòpez-De Fede A., Stewart J.E., Hardin J.W., Mayfield-Smith K. (2016). Comparison of small-area deprivation measures as predictors of chronic disease burden in a low-income population. Int. J. Equity Health.

[35] Doebler S., Glasgow N. (2016). Relationships between Deprivation and the Self-Reported Health of Older People in Northern Ireland. J. Aging Health.

[36] Whynes D.K. (2009). Deprivation and self-reported health: Are there “Scottish effects” in England and Wales?. J. Public Health.

[37] Yamaguchi A. (2015). Influences of Quality of Life on Health and Well-Being. Soc. Indic. Res..

[38] Cabrera-Barona P., Blaschke T., Kienberger S. (2017). Explaining Accessibility and Satisfaction Related to Healthcare: A Mixed-Methods Approach. Soc. Indic. Res..

[39] Allik M., Brown D., Dundas R., Leyland A.H. (2016). Developing a new small-area measure of deprivation using 2001 and 2011 census data from Scotland. Health Place.

[40] Carmone F.J., Kara A., Zanakis S.H. (1997). A Monte Carlo investigation of incomplete pairwise comparison matrices in AHP. Eur. J. Oper. Res..

[41] Miilunpalo S., Vuori I., Oja P., Pasanen M., Urponen H. (1997). Self-rated health status as a health measure: The predictive value of self-reported health status on the use of physician services and on mortality in the working-age population. J. Clin. Epidemiol..

[42] Bailis D.S., Segall A., Chipperfield J.G. (2003). Two views of self-rated general health status. Soc. Sci. Med..

